# Measurements of peri-prostatic adipose tissue by MRI predict bone metastasis in patients with newly diagnosed prostate cancer

**DOI:** 10.3389/fonc.2024.1393650

**Published:** 2024-04-26

**Authors:** Bo-Hao Liu, Yun-Hua Mao, Xiao-Yang Li, Rui-Xiang Luo, Wei-An Zhu, Hua-Bin Su, Heng-Da Zeng, Chu-Hao Chen, Xiao Zhao, Chen Zou, Yun Luo

**Affiliations:** ^1^ Department of Urology, The Third Affiliated Hospital of Sun Yat-sen University, Guangzhou, China; ^2^ Department of Urology, Kashgar First People’s Hospital, Kashgar, Xinjiang, China

**Keywords:** prostate cancer, metastasis, peri-prostatic adipose tissue, nomogram, MRI

## Abstract

**Objectives:**

To investigate the role of MRI measurements of peri-prostatic adipose tissue (PPAT) in predicting bone metastasis (BM) in patients with newly diagnosed prostate cancer (PCa).

**Methods:**

We performed a retrospective study on 156 patients newly diagnosed with PCa by prostate biopsy between October 2010 and November 2022. Clinicopathologic characteristics were collected. Measurements including PPAT volume and prostate volume were calculated by MRI, and the normalized PPAT (PPAT volume/prostate volume) was computed. Independent predictors of BM were determined by univariate and multivariate logistic regression analysis, and a new nomogram was developed based on the predictors. Receiver operating characteristic (ROC) curves were used to estimate predictive performance.

**Results:**

PPAT and normalized PPAT were associated with BM (P<0.001). Normalized PPAT positively correlated with clinical T stage(cT), clinical N stage(cN), and Grading Groups(P<0.05). The results of ROC curves indicated that PPAT and normalized PPAT had promising predictive value for BM with the AUC of 0.684 and 0.775 respectively. Univariate and multivariate analysis revealed that high normalized PPAT, cN, and alkaline phosphatase(ALP) were independently predictors of BM. The nomogram was developed and the concordance index(C-index) was 0.856.

**Conclusions:**

Normalized PPAT is an independent predictor for BM among with cN, and ALP. Normalized PPAT may help predict BM in patients with newly diagnosed prostate cancer, thus providing adjunctive information for BM risk stratification and bone scan selection.

## Introduction

Prostate cancer (PCa) remains the second most common male malignancy worldwide, and its incidence in China has been growing for the past few decades (1, 2). Bone metastasis (BM) was the prominent metastatic event in PCa, which was regarded as an adverse factor for survival and quality of life (3, 4). Currently, bone scan including single-photon emission computed tomography and computed tomography (SPECT/CT) has been widely used for diagnosing BM of PCa, with 92% sensitivity and 95% specificity (5–7). The incidence of BM is 11%-27% in PCa patients at the first diagnosis in China, and even lower in European and American countries (8, 9). Therefore, approximately three quarters of patients endured the examination costs and unnecessary radiation exposure. Additionally, many primary hospitals lack the relevant nuclear medicine equipment to carry out the examination. Some guidelines and reports appealed that not all patients should undergo bone scan but are only appropriate for patients with certain qualifications (5, 10, 11). However, these qualifications only considered clinicopathologic factors while lacking the radiological features. Therefore, there is a need to develop a risk stratification tool involving radiological parameters to improve BM prediction.

Increasing evidence revealed the close correlation between adiposity and PCa, but the body mass index (BMI) used in many studies can only represent generalized obesity of the whole body rather than the peri-organ distribution of adipose tissue (12, 13). Peri-prostatic adipose tissue (PPAT) is a special fat reservoir surrounding the prostate. More and more evidence shows that PPAT can not only store lipids, but also secrete a variety of factors (such as leptin, adiponectin, TNF-α, CCL7, etc.) that affect the progression of PCa (14–16). Previous studies demonstrated that PPAT accumulation is significantly correlated with higher tumor stage, higher Gleason scores, poorer prognosis, and local metastasis, showing promising predictive value of PCa aggressiveness (17–19). However, little is known on the relationship between PPAT accumulation and distant metastasis of prostate cancer, such as BM and visceral metastasis.

Numerous imaging techniques such as computerized tomography (CT), ultrasonography, and magnetic resonance imaging (MRI) have been applied to calculate PPAT accumulation. Ultrasonic waves would be attenuated by tissue and generate poor image quality, while CT might neglect the subcentimeter region of fat (20, 21). With superior depiction of fat border and image details, MRI was regarded as the highest resolution in detecting adipose tissue (22). Multiple Imaging modalities including thickness, area, and volume are used to represent PPAT accumulation (23), while the index of thickness and area are easy to measure but it could not show the accurate volume of PPAT, not to mention the tridimensional PPAT distribution. Therefore, PPAT volume is the most accurate method to represent PPAT accumulation. In this study, we aim to explore the association between MRI-measured PPAT characteristics and BM. Moreover, We built a predictive nomogram based on the clinicopathologic parameters and normalized PPAT to help patients for risk stratification and selection of bone scan.

## Materials and methods

### Patient selection and data collection

This study was approved by the Institutional Review Committee of the Third Affiliated Hospital of Sun Yat-sen University (NO [2022]:02-313-01). In this retrospective study, we analyzed data from 156 patients newly diagnosed with prostate cancer in the Department of Urology at the Third Affiliated Hospital of Sun Yat-Sen University between October 2010 and November 2022. All patients received technetium Tc 99m methylene diphosphonate single-photon emission computed tomography/computed tomography (Tc-99m-MDP SPECT/CT) after diagnosed by biopsy. They all had complete clinicopathologic data, including age, BMI, Grading Groups, cT, cN, PSA, ALP, serum calcium, serum phosphorus, complications and prostate MRI. Radiologists diagnosed BM by analyzing tracer uptake and imaging features in Tc-99m-MDP SPECT/CT. The final reports were confirmed and published by the Department of Nuclear Medicine of the Third Affiliated Hospital of Sun Yat-sen University. Grading Groups followed the ISUP (Internal Society of Urologic Pathology) consensus on Gleason grading of PCa, which were documented as the highest Gleason score in all biopsy cores (24). Patients with ambiguous bone scan results, malignant diseases of non-prostatic origin, a history of prostate surgery or after any neoadjuvant therapy were excluded.

### PPAT measurement

All patients routinely received 1.5T or 3.0T MRI examination at our hospital which was taken within 3 months before prostate biopsy. Regions of interest (ROI) were measured by using 3D Slicer software(5.2.1) on axial T2-weighted MRI images. All measurements of ROI were performed two times by two urologists who were trained by the radiologist, with no knowledge of clinical and pathological information. The mean values measured by two urologists were used for statistical analysis. Intraclass correlation coefficient (ICC) was used to test inter-observer reliability.

The PPAT is defined as the adipose tissue surrounding the prostate, with the lateral boundary being the first visible fascia adjacent to the levator muscle, the posterior boundary being the Denonville fascia, and the anterior boundary being the symphysis pubis (19). We measured continuous T2-weighted MRI images from the apex to the base of prostate, PPAT and prostate volume were calculated by using the volume formula = the sum of contour area × slice thickness. Normalized PPAT was defined as dividing PPAT volume by the prostate volume.

### Statistical analysis

The mean (Standard Deviation[SD]) or median (Interquartile Range[IQR]) was used to represent continuous data. Comparisons were made using the student t test or Mann-Whitney test. Categorical variables were compared using Chi-square test or Fisher’s exact test, depending on the case. Part of the variables, including ALP (≤120U/L vs. >120U/L), cT (≤2 vs. >2), Grading Groups (≤3 vs. >3), PSA (≤10 ng/mL vs. 10-20 ng/mL vs. >20 ng/mL), were converted to categorical variables or regrouped in order to facilitate the analysis and model design. Spearman correlation test was used to analyze the correlation between normalized PPAT and other variables. The independent risk factors of BM were determined by univariate and multivariate logistic regression analysis. ROC curves were performed to calculate the best cut-off values of normalized PPAT, and the area under the curve (AUC) and 95% confidence interval (CI) were calculated. A new nomogram was generated based on the results of the multivariate logistic regression analysis to predict BM. The assessment methods of the nomogram include C-index and calibration curves. All statistical analyses were performed with SPSS 26.0 and R version 4.3.1, and *P* value <0.05 was considered statistically significant.

## Results

### Patient characteristics

A total of 156 patients were included in this study, of which 114 were diagnosed without BM and 42 were diagnosed with BM (26.9%). The demographic, clinicopathologic, and PPAT measurements for all patients were shown in **Table 1**. The median age and BMI were 70 years and 23.19 kg/m^2^, respectively. The statistical analysis showed that ALP (*P <*0.001), PPAT volume (*P <*0.001), normalized PPAT (*P <*0.001), PSA (*P* =0.024), cT (*P <*0.001), cN (*P <*0.001) and Grading Groups (*P* =0.010, were significantly different between the patients in the two groups, whereas age (*P* =0.186), BMI (*P* =0.734), serum calcium (*P* =0.847), serum phosphorus (*P* =0.873), prostate volume (*P* =0.981), diabetes (*P* =0.193), hypertension (*P* =0.942) and coronary heart disease (*P* =0.972) were not statistically significant. The median prostate volume and PPAT volume were 37.73 cm³ and 29.66 cm³, respectively. Excellent reproducibility of measurement was determined using ICC and assessed as 0.986 for PPAT volume (*P <*0.001) and 0.995 for prostate volume (*P <*0.001).

**Table 1:** Demographic, clinicopathologic, and PPAT data for all patients.

Variables	Total (n=156)	Non-BM (n=114)	BM (n=42)	P*	Comparison methods
Age (years), mean ± SD	70 ± 8	71 ± 8	69 ± 9	0.186	student t test
BMI (kg/m2), mean ± SD	23.19 ± 3.41	23.14 ± 3.50	23.35 ± 3.16	0.734	student t test
ALP (U/L), median (IQR)	69.00 (55.00-84.00)	64.00 (53.50-77.25)	84.50 (65.50-156.00)	**<0.001**	Mann-Whitney test
Serum calcium (mmol/L), mean ± SD	2.33 ± 0.11	2.33 ± 0.11	2.33 ± 0.14	0.847	student t test
Serum phosphorus (mmol/L), median (IQR)	1.09 (0.99-1.20)	1.08 (0.99-1.21)	1.11 (1.00-1.17)	0.873	Mann-Whitney test
Prostate volume (cm3), median (IQR)	37.73 (27.82-53.12)	38.82 (27.05-52.29)	35.55 (28.95-54.02)	0.981	Mann-Whitney test
PPAT volume (cm3), median (IQR)	29.66 (18.56-43.99)	26.32 (15.35-36.83)	42.55 (23.20-60.83)	**<0.001**	Mann-Whitney test
Normalized PPAT, median (IQR)	0.6885 (0.5341-1.0041)	0.6450 (0.5100-0.8425)	1.05 (0.7200-1.3725)	**<0.001**	Mann-Whitney test
PSA (ng/mL), n (%)				**0.024**	Chi-square test
≤10	20	18 (15.8)	2 (4.8)		
10<PSA ≤ 20	40	33 (28.9)	7 (16.7)		
PSA>20	96	63 (55.3)	33 (78.5)		
ALP (U/L), n (%)				**<0.001**	Chi-square test
≤120	139	111 (97.4)	28 (66.7)		
>120	17	3 (2.6)	14 (33.3)		
cT, n (%)				**<0.001**	Chi-square test
T1-T2	91	78 (68.4)	13 (31)		
T3-T4	65	36 (31.6)	29 (69)		
cN, n (%)				**<0.001**	Chi-square test
N0	96	84 (73.7)	12 (28.6)		
N1	60	30 (26.3)	30 (71.4)		
Grading Groups (ISUP), n (%)				**0.010**	Chi-square test
≤3	51	44 (38.6)	7 (16.7)		
>3	105	70 (61.4)	35 (83.3)		
Diabetes, n (%)				0.193	Chi-square test
No	127	90 (78.9)	37 (88.1)		
Yes	29	24 (21.1)	5 (11.9)		
HTN, n (%)				0.942	Chi-square test
No	101	74 (64.9)	27 (64.3)		
Yes	55	40 (35.1)	15 (35.7)		
CHD, n (%)				0.972	Chi-square test
No	126	92 (80.7)	34 (81)		
Yes	30	22 (19.3)	8 (19)		

*student t test; Mann-Whitney test; Chi-square test; P values in bold are indicative of statistical significance(<0.05). SD, Standard Deviation; IQR, Interquartile Range; BMI, Body Mass Index; HTN, Hypertension; CHD, Coronary Heart Disease; ALP, Alkaline Phosphatase; PSA, Prostate Specific Antigen; PPAT, Peri-Prostatic Adipose Tissue; cT, Clinical T stage; cN, Clinical N stage; ISUP, International Society of Urological Pathology.

**Figure 1:**
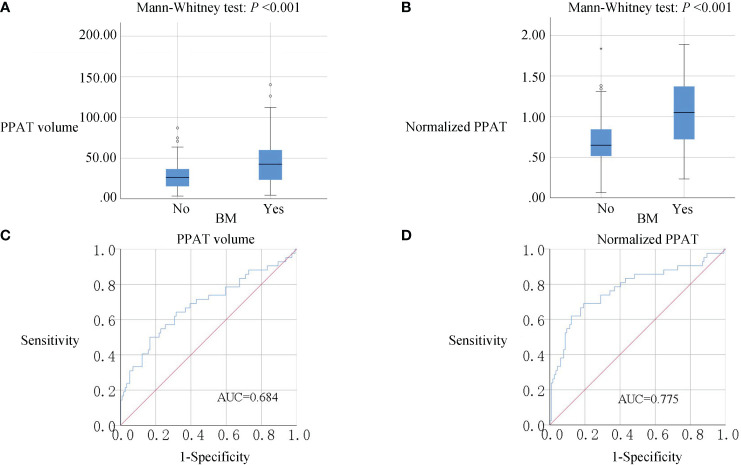
**(A, B),** Box plots of PPAT measurements. PPAT volume and normalized PPAT in BM group were significantly higher than non-BM (P<0.001). **(C, D)**, ROC analysis of PPAT volume and normalized PPAT for the prediction of BM. AUC: 0.684 for PPAT volume and 0.775 for normalized PPAT.

As is shown in **Figure 1**, PPAT volume in the BM group[42.55(23.20-60.83)] was significantly higher than Non-BM group[26.32(15.35-36.83)](*P <*0.001) (**Figure 1A**). Normalized PPAT in BM group[1.05(0.7200-1.3725)] was significantly higher than Non-BM group[0.6450(0.5100-0.8425)](*P <*0.001) (**Figure 1B**). ROC curves analyses revealed that high PPAT volume and high normalized PPAT were associated with BM(*P <*0.001) (**Figure 1C, D**). We compared the ability to predict BM by PPAT volume and normalized PPAT measurements and found high normalized PPAT[ROC AUC (95% CI) 0.775 (0.683, 0.867), *P <*0.001] was better than high PPAT volume[ROC AUC (95% CI) 0.684 (0.581, 0.786), *P <*0.001]. Therefore, we included normalized PPAT in the model. We used the best cut-off values of normalized PPAT (0.894) to stratify patients into the high group (normalized PPAT≥0.894, n = 51) and low group (normalized PPAT <0.894, n = 105).

**Figure 2:**
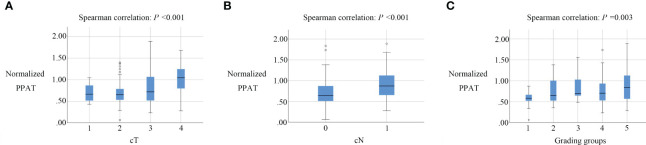
Box plots of normalized PPAT in prostate cancers with different cT, cN, Grading groups.Normalized PPAT had a significantly correlation with cT **(A)**, cN **(B)**. and Grading groups **(C)**.

**Table 2** showcases the findings regarding the correlation between normalized PPAT and other factors. Normalized PPAT was significantly correlated with cT (ρ= 0.319, *P* <0.001), cN(ρ= 0.294, *P* <0.001), and Grading Groups(ρ= 0.238, *P* =0.003) (**Figure 2**). Meanwhile, no significant correlation was found between normalized PPAT and other factors. Additionally, no multicollinearity relationship existed between each factors.

**Table 2:** Correlation analysis of normalized PPAT.

	Normalized PPAT	
	Coefficient	P
Age,years	0.020	0.805
BMI,kg/m2	-0.070	0.383
PSA,ng/mL	0.123	0.126
ALP,U/L	0.140	0.081
Serum calcium,mmol/L	0.029	0.720
Serum phosphorus,mmol/L	0.026	0.751
cT	0.319	**<0.001**
cN	0.294	**<0.001**
Grading Groups(ISUP)	0.238	**0.003**
Diabetes	-0.061	0.450
HTN	0.138	0.087
CHD	-0.064	0.428

The Spearman correlation test was used to analyse the correlation between normalized PPAT and clinicopathologic factors. P values in bold are indicative of statistical significance(<0.05). BMI, Body Mass Index; HTN, Hypertension; CHD, Coronary Heart Disease; ALP, Alkaline Phosphatase; PSA, Prostate Specific Antigen; PPAT, Peri-Prostatic Adipose Tissue; cT, Clinical T stage; cN, Clinical N stage; ISUP, International Society of Urological Pathology.

### Logistic regression and nomogram development

**Table 3** showed the results of univariate and multivariate logistic regression analysis. Univariate analysis showed that cT(OR: 4.833, 95%CI: 2.251-10.378, *P* <0.001), cN(OR: 7.000, 95%CI: 3.181-15.403, *P* <0.001), ALP(OR: 18.500, 95%CI: 4.972-68.841, *P* <0.001), Grading Groups(OR: 3.143, 95%CI: 1.284-7.690, *P* =0.012), PSA(OR: 4.714, 95%CI: 1.031-21.563, *P* =0.046), and normalized PPAT(OR: 9.329, 95%CI: 4.180-20.817, *P* <0.001) were significant favorable predictors. Subsequently, multivariate analysis with all of the above clinically relevant variables showed that high normalized PPAT(OR: 4.928, 95%CI: 1.910-12.713, *P* =0.001), cN(OR: 4.424, 95%CI: 1.674-11.693, *P* =0.003), and ALP(OR: 11.743, 95%CI: 2.153-64.045, *P* =0.004) were independent risk factors for BM.

**Table 3:** Univariate and multivariate analyses of potential factors predicting bone metastasis in all patients.

		Univariate analysis		Multivariate analysis	
Variables		OR (95%CI)	P	OR (95%CI)	P
Age,years		0.971 (0.93-1.014)	0.186		
BMI,kg/m^2^		1.018 (0.918-1.13)	0.732		
Serum calcium,mmol/L		1.358 (0.062-29.686)	0.846		
Serum phosphorus,mmol/L		1.653 (0.241-11.323)	0.609		
Diabetes	no	–			
	yes	0.507 (0.180-1.429)	0.199		
HTN	no	–			
	yes	1.028 (0.491-2.152)	0.942		
CHD	no	–			
	yes	0.984 (0.400-2.420)	0.972		
cT					
	≤2	–			
	>2	4.833 (2.251-10.378)	**<0.001**	1.526 (0.569-4.093)	0.402
cN					
	0	–			
	1	7.000 (3.181-15.403)	**<0.001**	4.424 (1.674-11.693)	**0.003**
ALP,U/L					
	≤120	–			
	>120	18.500 (4.972-68.841)	**<0.001**	11.743 (2.153-64.045)	**0.004**
Grading Groups (ISUP)					
	≤3	–			
	>3	3.143 (1.284-7.690)	**0.012**	1.126 (0.333-3.815)	0.848
PSA,ng/mL					
	≤10	–			
	10<PSA ≤ 20	1.909 (0.358-10.173)	0.449		
	PSA>20	4.714 (1.031-21.563)	**0.046**	2.775 (0.479-16.074)	0.255
Normalized PPAT					
	Low	–			
	High	9.329 (4.180-20.817)	**<0.001**	4.928 (1.910-12.713)	**0.001**

OR=Odds Ratio, CI=Confidence Interval. P values in bold are indicative of statistical significance(<0.05). BMI, Body Mass Index; HTN, Hypertension; CHD, Coronary Heart Disease; ALP, Alkaline Phosphatase; PSA, Prostate Specific Antigen; PPAT, Peri-Prostatic Adipose Tissue; cT, Clinical T stage; cN, Clinical N stage; ISUP, International Society of Urological Pathology.

**Figure 3:**
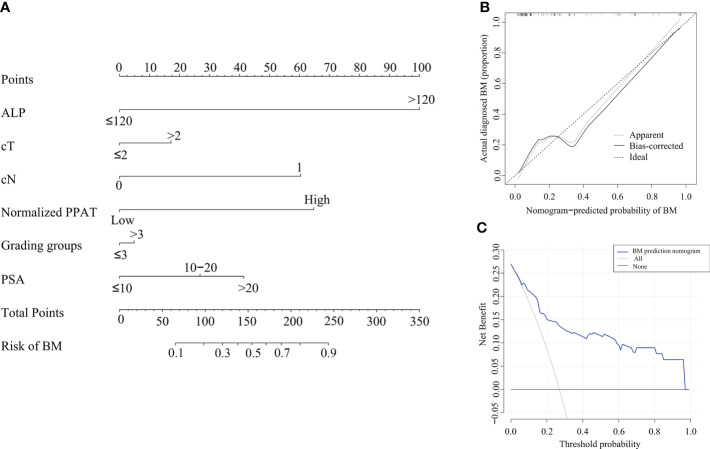
Nomogram to predict BM in first diagnosed PCa patients. **(A)**, Nomogram based on predictors for BM. By drawing a line straight upward to the point axis, each factor corresponds to a specific point. After adding the points on the total points axis, the probability of BM will represent by drawing a straight line down to the risk axis. **(B)**, Calibration curves of the nomogram prediction. The X-axis represents the predicted probability and the Y-axis represents the actual probability. The diagonal dotted line represents the perfect prediction of the ideal model and the solid line represents the performance of the nomogram. **(C)**, Decision curve analysis: the Y-axis measures the net benefit. The blue line represents the nomogram. The black and thin gray lines represent the assumption that all patients have BM and no patients have BM, respectively.

Based on the results of multivariate analysis, we created a nomogram (**Figure 3A**) to predict BM, including high normalized PPAT, cN, and ALP. Although the Grading Groups, PSA and cT were not independent risk factors for BM in our study, these factors were clinically relevant to PCa aggressiveness, thus they were incorporated in the nomogram. The C-index of this nomogram was 0.856. **Figure 3B, C** showed the calibration curve and decision curve.

## Discussion

PCa shows a tendency toward metastasizing to the bone. BM significantly influences patients’ treatments and prognosis (25). To date, Bone scan has been widely used diagnostic method and the most frequent examination for BM diagnosis (5).Combined with whole-body bone single-photon emission computed tomography and computed tomography, SPECT/CT aids in reducing superimposition from activity, thereby leading to enhanced anatomical localization and a clearer distinction ability, which proves to be a more precise technique in comparison to planar bone scan (6, 7). Although with high precision in diagnosing BM, not all PCa patients benefit from bone scan. Only 11%-27% of PCa patients in China, and approximately 3% in European countries and the United States were diagnosed with BM at the first bone scan (8, 9, 26, 27). More than half of newly diagnosed PCa patients do not need regular bone scan, which caused patients’ extra financial burden. In addition, unnecessary bone scans increased the risk of radiation exposure, which is thought to increase the risk of various malignant diseases and radiation-induced cataracts (28). Therefore, it is necessary to assess the risk of BM in patients with PCa and to predict whether the patient needs a bone scan.

The clinicopathologic characteristics of cT, biopsy Gleason source(BGS), and ALP were widely used to predict the risk of BM and developed standards for bone scan. Briganti et al. stated that patients with a BGS ≤7 or PSA ≤10 ng/ml and cT<2 might be safely omitted bone scan (26). McArthur et al. stated that for newly diagnosed PCa patients with a BGS <8 and a tPSA <20 ng/ml, a bone scan could be safely omitted, and the standard had a negative predictive value of 100% (29). Liu et al. researched 322 Chinese patients and demonstrated that a bone scan is only recommended for patients with PSA >39.58 ng/ml, prostate specific antigen density(PSAD) >1.489 ng/(ml/cm^3^), ALP >91.0 U/l and BGS >7.5 (30). Chen et al. analyzed 308 Chinese patients and built four models according to different distinctions between the cT, BGS, tPSA, and ALP. Finally, they found that the model had the highest predictive values when the grouping scheme of cT was cT1-cT2 and cT3-cT4, BGS was ≤7 and 8-10, and ALP was ≤120U/L and > 120U/L. And the patients who meet the following conditions should be highly suspected of having BM, when the clinical stage is cT1-cT2, the clinicopathologic indicators should meet the requirements of BGS ≤7, ALP >120 U/L and tPSA >90.64ng/ml or BGS ≥8, and ALP >120 U/L; when the clinical stage is cT3-cT4 clinicopathologic indicators should meet the requirements of BGS ≤7, and ALP >120 U/L or BGS ≥8 (31). However, these studies only considered clinicopathologic factors while lacking the characteristics of imaging.

Prostate is wrapped in a special reservoir of fat called PPAT, which stores lipids and possesses vigorous metabolic activity. One-third of PPAT is in direct contact with the prostate tissue and might increase the aggressiveness PCa and promote local dissemination (32). Several mechanisms have been reported to interpret the roles of PPAT in the development of PCa, including the paracrine and endocrine of various growth factors, the inducement of lipolysis, the stimulation of chronic inflammation, and the adjustment of chemokine pathways (14, 16, 33, 34). PPAT accumulation was regarded as a predictive factor for the aggressiveness, prognosis, metastasis, and response to hormone treatment of PCa. Ultrasound, CT, and MRI images were used to measure the three main indexes of PPAT including thickness, area, and volume in different studies. Woo et al. measured PPAT thickness of 190 patients which defined as the shortest perpendicular distance from pubis symphysis to skin and prostate (17). They found a positive correlation between the PPAT thickness and BGS. Zhai et al. selected an axial section at the junction of the bladder and prostate at the level of the pubis symphysis to represent PPAT (18). They also calculated a normalized index by dividing the PPAT area by the prostate area called PPFA/PA. According to the report, PPFA/PA is closely associated with PCa aggressiveness and is an independent predictive factor for lymph node metastasis. While the index of thickness and the single image are easy to measure but it could not show the accurate volume of PPAT not to speak the tridimensional distribution. Due to the operator subjectivity and depth-dependent attenuation of ultrasound and the poor resolution ratio of adipose tissue on CT, MRI measured PPAT and prostate volume on consecutive images was regarded as the most accurate method. Normalized PPAT was defined as dividing PPAT volume by the prostate volume, in order to alleviate the influence of prostate volume on PPAT volume. Previous studies have demonstrated these two parameters were associated with biochemical recurrence, Grading groups progression, and the time to castration resistant prostate cancer (19, 23, 35–37). However, little is known about its potential role in predicting PCa bone metastasis.

This study revealed the close relationship between PPAT measurements especially normalized PPAT and PCa aggressiveness in accordance with previous studies. In addition, we found that PPAT volume and normalized PPAT in BM patients were significantly higher than those patients without BM. In order to further investigate the predictive performance of PPAT volume and normalized PPAT, ROC analysis was performed and found that normalized PPAT had a superior AUC value(0.775). In 42 (26.9%) PCa patients with BM in our study, we first proved that normalized PPAT could serve as an independent predictor for BM in first-diagnosed PCa patients. Finally, we developed a nomogram for predicting BM in first-diagnosed PCa patients. And this nomogram included six predictors due to the conclusion of the multivariate analysis and abundant clinical utilities: normalized PPAT, cT, cN, ALP, PSA, and Grading groups. Our new nomogram was able to accurately predict the risk of BM in first-diagnosed PCa patients, as tested by C-index (0.856), calibration curve and decision curve analysis.

Due to the criteria being varied in different guidelines, when converting the clinicopathologic parameters and normalized PPAT into categorical variables, the standards of thresholds were based on previous studies and the best cut-off values of the ROC curve (31). According to the research report, Epigenome-wide DNA methylation profiling of PPAT may cause changes in lipid metabolism, immune dysregulation, and adverse PCa microenvironment, thus promoting PCa metastasis (38). Wang et al. found that those PCa patients with BM had elevated total levels of free fatty acids and caprylic acid (C8:0), which may promote the differentiation of bone mesenchymal stem cells derived adipocytes and finally promote the invasion and migration of PCa (39). Nevertheless, the effect of those acids in PPAT is still unclear. Therefore, the potential mechanisms of how PPAT accumulation promotes bone metastasis need further investigation.

Furthermore, the utilization of imaging features and radiomics has been applied in several studies to evaluate the aggressiveness of PCa, which was considered as a reflection, to some degree, of the tumor immune microenvironment. Tafuri et al. conducted a study in which they recorded and analyzed the apparent diffusion coefficient (ADC) of PPAT using MRI. They found that lower ADC values of PPAT are linked to higher biopsy ISUP grading groups and a greater percentage of positive cores in prostate biopsy (40). Shahait et al. identified six radiomic features of PPAT from MRI that can potentially predict patients with clinically significant PCa (41). However, the specific associations of imaging features with PCa immune microenvironment and its predictive value for metastasis require further investigation.

Objectively, our study had some limitations. First of all, it was a retrospective study based on 156 Chinese patients in a single medical center. Our findings need further verification in different regions and ethnic groups. Secondly, because of the technical and manpower limitation, we can only analyze the spatial characteristics of PPAT through manual measurement of continuous images by urologists, even if they were trained by expert radiologists. Automatic PPAT segmentation based on artificial intelligence may provide a time-saving method with satisfactory accuracy (42). Thirdly, prostate-specific membrane antigen positron emission tomography/computed tomography(PSMA PET/CT) is a new diagnostic tool with better sensitivity and specificity than SPECT/CT, which was regarded as the most accurate noninvasive method for diagnosing BM (6, 43). Given the high cost and the high level of radiation exposure, the criteria for which subgroup of patients should be examined PSMA PET/CT need to be further explored.

## Conclusions

Normalized PPAT is closely associated with cT, cN, Grading groups, and bone metastasis in first-diagnosed PCa patients. And high normalized PPAT serves as an independent predictor for BM. This new nomogram can provide adjunctive information for BM risk stratification and help determine if a bone scan is needed.

## Data availability statement

The raw data supporting the conclusions of this article will be made available by the authors, without undue reservation.

## Ethics statement

The studies involving humans were approved by the Institutional Review Committee of the Third Affiliated Hospital of Sun Yat-sen University. The studies were conducted in accordance with the local legislation and institutional requirements. Written informed consent for participation was not required from the participants or the participants’ legal guardians/next of kin in accordance with the national legislation and institutional requirements.

## Author contributions

BHL: Conceptualization, Data curation, Formal analysis, Methodology, Visualization, Writing – original draft, Writing – review & editing. YHM: Conceptualization, Data curation, Formal analysis, Writing – original draft, Writing – review & editing. XYL: Conceptualization, Data curation, Formal analysis, Methodology, Writing – review & editing. RXL: Methodology, Software, Visualization, Writing – review & editing. WAZ: Conceptualization, Data curation, Writing – review & editing. HBS: Conceptualization, Data curation, Writing – review & editing. HDZ: Data curation, Writing – review & editing. CHC: Data curation, Writing – review & editing. XZ: Data curation, Writing – review & editing. CZ: Data curation, Writing – review & editing. YL: Funding acquisition, Investigation, Project administration, Resources, Supervision, Validation, Writing – original draft, Writing – review & editing.
